# Identification of the parasitoid community associated with an outbreaking gall wasp, *Zapatella davisae*, and their relative abundances in New England and Long Island, New York

**DOI:** 10.1002/ece3.4543

**Published:** 2018-12-13

**Authors:** Monica J. Davis, Jeremy C. Andersen, Joseph Elkinton

**Affiliations:** ^1^ Department of Environmental Conservation University of Massachusetts Amherst Massachusetts; ^2^ Present address: Department of Environmental Science Policy and Management University of California Berkeley Berkeley California

**Keywords:** biological control, Cynipidae, diversity, management, natural enemies, parasitoid community

## Abstract

Gall wasps (Hymenoptera: Cynipidae) are phytophagous insects that often go unnoticed; however, when they are introduced to a new area or released from their natural enemies, they have the capacity to outbreak and cause extensive foliar damage. One such outbreaking pest, *Zapatella davisae *(Cynipidae: Cynipini), causes significant damage and mortality to black oak, *Quercus velutina*, in the northeastern United States. In this study, we aimed to identify the parasitoid community associated with *Z. davisae*, compare differences in percent parasitism of *Z. davisae* in Cape Cod and Long Island, and determine which parasitoid species contribute most to parasitism in each region. From both locations, we reared parasitoids, identified morphological groups, analyzed percent parasitism rates for each group, and used DNA barcoding to provide species‐level identifications. On Long Island, there was nearly 100% parasitism in 2015 followed by a near total collapse of the population in 2016. In contrast, parasitism rates were lower and remained consistent on Cape Cod between 2015 and 2016, which may explain the greater canopy damage observed in that region. Species of Sycophila were the dominant parasitoids, with one species *Sycophila *nr. *novascotiae* representing ~65% of reared parasitoids from Long Island, and two species of *Sycophila *(*S. *nr. *novascotiae *and *S. foliatae*) with near equal representations on Cape Cod. In order to manage an insect pest, it is important to understand factors that influence its mortality and survival. An understanding of how these infestations progress overtime can help predict the impact that newer infestations in Nantucket, MA, and coastal Rhode Island will have on black oak populations and will aid in the management of this rapidly spreading gall wasp pest.

## 1 INTRODUCTION

Due to changes in global climate patterns and/or other anthropogenic disturbances, many indigenous species are expanding their ranges and establishing in novel ecosystems (Logan, Regniere, & Powell, 2003; Prior & Hellmann, 2013; Pyšek & Davidson, 2010). Most native insect herbivores have little impact on the host plants upon which they feed, due to the suppression of their population densities by natural enemies or toxic plant chemicals (i.e. top‐down or bottom‐up control, respectively; Keane & Crawley, 2002; Rosenheim, 1998). However, a native insect herbivore may outbreak in a novel ecosystem, as a result of a time lag between the range expansion of the herbivore and the re‐establishment of its community of parasitoids and predators (Didham, Tylianakis, Hutchison, Ewers, & Gemmell, 2005; Sanders, Gotelli, Heller, & Gordon, 2003).

One group of herbivores that has been known to experience occasional outbreaks following range expansion (Schönrogge, Stone, & Crawley, 1995) is the oak gall wasps (Hymenoptera: Cynipidae). Oak gall wasps form galls on various tissues, including stems, leaves and fruits, (Raman, Schaefer, & Withers, 2011) that provide food and protection for the developing gall maker (Ronquist et al., 2015), as well as a diverse community of insects (Stone, Schönrogge, Atkinson, Bellido, & Pujade‐Villar, 2002). Members of these communities include herbivores, parasitoids, and inquilines (Ito & Hijii, 2004; Ronquist, 1994). Interactions between oak gall wasps and parasitoids always result in the death of one of the two participants (i.e. either the gall wasp is consumed by the developing parasitoid larvae, or the gall wasp mounts an immune response and encapsulates the parasitoid egg before it can develop). In contrast, inquilines—phytophagous insects that develop within a gall, but are incapable of producing their own—can have fatal or nonfatal interactions with gall wasps (Stone et al., 2002). As such, parasitoids are often considered sources of direct mortality and inquilines as sources of indirect mortality (Stone et al., 2002). These direct and indirect sources of mortality have the potential to regulate populations of native oak gall wasps (Otake, Moriya, & Shiga, 1984; Schönrogge, Stone, & Crawley, 1996a, 1996b ; Stone et al., 2002; Washburn & Cornell, 1979); however, it is possible for outbreaks to occur during parasitoid–predator lag times in newly invaded regions (e.g. Schönrogge et al., 1995).

Recently, extensive tree mortality and damage (flagging, leaf clumping, and limb loss) has been documented for black oaks (*Quercus velutina* Lamarck [Fagales: Fagaceae]), in Long Island, New York, and coastal Rhode Island, as well as Cape Cod, Martha's Vineyard, and Nantucket, Massachusetts. A newly described gall wasp species, *Zapatella davisae *Buffington (Hymenoptera: Cynipidae; Buffington, Melika, Davis, & Elkinton, 2016), has been identified as the cause of this damage. *Zapatella davisae* larvae develop under the bark in late summer, and adults emerge in early spring (Buffington et al., 2016). Widespread mortality and defoliation of black oak were first documented on Long Island in 1990, and subsided five years later. The reason for this population crash is still unknown (Pike, Robinson, & Abrahamson, 2001). More recently, outbreaks were reported north of Long Island on Cape Cod and Martha's Vineyard in 2008 (Davis, Elkinton, & Norton, 2017). Our field surveys have shown that while *Z. davisae* infests the same proportion of trees in Long Island and Cape Cod, it causes significantly more canopy damage on Cape Cod than Long Island (Davis et al., 2017). Differences in levels of infestation between the two regions suggest that some factor may be differentially regulating densities of these two populations.

Given previous examples of oak gall wasps expanding their distribution and escaping from their native parasitoids (e.g. Cooper & Rieske, 2007; Eliason & Potter, 2000; Pujade‐Villar, Cibrian‐Tovar, Barrera‐Ruiz, & Melika, 2014; Schönrogge et al., 1995), we were curious as to whether discrepancies in *Z. davisae's* parasitoid communities may correlate with variable damage to black oak on Cape Cod and Long Island. Therefore, our objectives were to (a) identify the parasitoid community associated with *Z. davisae*; (b) compare differences in percent parasitism of *Z. davisae* in Cape Cod and Long Island; and (c) determine which parasitoid species contribute most to parasitism in each region.

## 2 MATERIALS AND METHODS

### 2.1 Sample collection

To collect inquilines and parasitoids of *Z. davisae*, black oak branches with visible stem gall formations were collected at four sites on Long Island and at four sites on Cape Cod in mid‐April 2015 and 2016, approximately two weeks before expected gall wasp emergence. At each site, three branches with galls were haphazardly collected from the upper portion of the canopy of 10 black oak trees, for a total of 120 samples per region each year (*n* = 480). Each branch included the present years’ growth and the previous years’ growth. Upon collection, each branch was placed in an individual Berlese funnel trap (BioQuip, Rancho Dominguez, CA) that was lined with black paper on the bottom half, so insects would be trapped in the top half after emergence. Occasionally, wasps fell into the bottom portion of the container. These specimens were still collected and recorded. Floral Foam Micro Bricks^®^ (Oasis^® ^Floral Products, Kent OH) were placed in the bottom of the 11.4 × 20 cm traps to keep branches alive. Trap containers were held at room temperature until gall wasp or parasitoid emergence was complete in early June (Buffington et al., 2016). Gall wasps, inquilines, and parasitoids were counted for each branch, and emerged specimens were preserved individually in 95% ethanol for molecular analysis.

Photographs were taken of all emerged insects under a dissecting microscope (Nikon SMZ1000, SPOT graphics), and specimens were sorted into community components (i.e. *Z. davisae*, inquilines, and parasitoids). Parasitoid specimens were sorted by family and then into morphologically similar groups (hereafter, “morpho‐groups”). While we reared both inquilines and parasitoids from galls, we concentrated only on the diversity and potential impacts of parasitoids, as parasitoid–gall wasp interactions always result in the mortality of one of the two participants and inquiline–gall wasp interactions can exist without the mortality of either participant (Stone et al., 2002). Over the course of the study, we removed 70 inquilines, four Aphelinidae, 20 Cecidomyiidae, four Chloropidae, and multiple thrip and midge species that are not associated with gall wasp parasitism.

### 2.2 DNA extraction and amplification

To obtain species‐level identifications for parasitoids collected in the rearing study, we extracted DNA from 3 to 5 individuals per parasitoid morpho‐group using the Qiagen DNeasy Blood & Tissue Kit (Qiagen Co.) following the manufacturer's protocol. Prior to cell lysing, samples were ground with a mortar and pestle, and after DNA was extracted, we amplified fragments of two target gene regions. The nuclear ribosomal gene 28S was amplified with the forward primer s3660 (5′‐GAGAGTTMAASAGTACGTGAAAC‐3′) and reverse primer 28b (5′‐TCGGAAGGAACCAGCTACTA‐3′) (Morse & Normark, 2006) according to the PCR protocol described in Morse and Normark (2006). For the mitochondrial gene cytochrome oxidase I (COI) gene, we amplified two nonoverlapping fragments. For most of the parasitoids we examined, we amplified a fragment of the 3′ region of COI using the forward primer Jerry (5′‐CAACATTTATTTTGATTTTTTGG‐3′) and reverse primer Pat (5′‐TCCAATGCACTAATCTGCCATATTA‐3′) (Ghararieh, Bruford, Dawah, Fernandes, & Dodd, 2006; Simon et al., 1994) with the PCR protocol described in Lotfalizadeh, Delvare, and Rasplus (2008). In addition, we amplified a fragment that corresponds with the “Barcode” region of COI using a novel forward primer “EurytomidF” (5′‐CCWGGKTCWTTAATTGGRAATGATC‐3′) and the commonly used barcode reverse primer HCO (Simon et al., 1994) using the PCR protocol described in Hebert, Penton, Burns, Janzen, and Hallwachs (2004). PCR products were visualized on 1.5% agarose gels, purified using Exonuclease 1 (Thermo Fisher Scientific) and Shrimp Alkaline Phosphatase (New England Biolabs) according to the Thermo Scientific PCR and purification protocol, and sequenced at the Yale Genomic Lab using an ABI 3730 sequencer (Life Technologies). Forward and reverse sequence reads were then aligned and edited using Geneious 8.1.8 (Kearse et al., 2012), and a consensus sequence was generated for each sample.

### 2.3 Species identification

After editing the consensus sequences for all parasitoids, sequences were aligned, manually adjusted, and truncated to the length of the shortest sequence using GENEIOUS (Kearse et al., 2012). To identify our parasitoid species, we compared our sequences to those published in the NCBI GenBank database using the blastn search algorithm (Altschul, Gish, Miller, Myers, & Lipman, 1990; Benson et al., 2013), as well as to the Barcode of Life Database (Ratnasingham & Hebert, 2013) using their “identification” algorithm. To differentiate putative species, we used a threshold of 3% variation between individuals for COI (Hebert et al., 2004) and 1% variation for 28S (Rubinoff, Cameron, & Will, 2006).

### 2.4 Statistical analyses

All statistical analyses were conducted in RStudio version 0.99.491 (Racine, 2011), an integrated development environment of the R programming software (R Core Team, 2015). At each location, parasitism rates were calculated as the number of emerged parasitoids divided by the total number of emerged parasitoids and gall wasps. Average percent parasitism was then compared between the two regions with a logistic regression, specifically a generalized linear model (GLM) with a binomial distribution. The average number of individuals (gall wasps and parasitoids) that emerged per branch was compared across regions and years using a Poisson model. To determine differences in the amount that each parasitoid species group contributed to parasitism, we conducted chi‐square tests to separately compare the counts of each parasitoid species group (delimited based on COI similarity thresholds) in each region for the 2015 and 2016 samples.

## 3 RESULTS

### 3.1 Species identification and sequence comparison

We collected 304 *Z. davisae* individuals, 653 parasitoids, and 70 inquilines over the course of this study. Prior to DNA extraction, the parasitoid samples were sorted into five distinct morphological groups. For 28S, we amplified a ~780 base pair fragment from three to five individuals from each morpho‐group. All morpho‐groups had high percentage matches to sequences published in GenBank (>98% for all morpho‐groups), though only morpho‐group *Sycophila* sp. 4 had a match of ≥99% to a sequence with a species‐level identification (JN623675.1 *Sycophila texana*; Table 1). For COI, we amplified a 645 base pair fragment using the primer pair Pat and Jerry from between two and six specimens from each morpho‐group. Unfortunately, none of the sequenced fragments had high percentage matches to published sequences (~90% for all morpho‐groups).

**Table 1:** GenBank top match hits for amplified fragments of COI and 28S from parasitoids reared from *Zapatella davisae*

Fragment	Specimen	Morpho‐group	NCBI	PIDENT	Species
COI	LIC5	*Sycophila* sp. 1	MH544116.1	100	*Sycophila globuli*
COI	LIC60	*Sycophila* sp. 1	MH544116.1	99.01	*Sycophila globuli*
COI	CCA1	*Sycophila* sp. 2	MH544108.1	100	*Sycophila flava*
COI	LIA115	*Sycophila* sp. 2	MH544105.1	100	*Sycophila flava*
COI	LIC4	*Sycophila* sp. 2	MH544105.1	100	*Sycophila flava*
COI	LIC62	*Sycophila* sp. 2	MH544105.1	99.6	*Sycophila flava*
COI	LIC23	*Sycophila* sp. 3	MH544143.1	100	*Sycophila *nr*. novascotiae*
COI	CCA97	*Sycophila* sp. 4	MH544119.1	99.4	*Sycophila foliatae*
COI	LIA104	*Sycophila* sp. 4	MH544119.1	100	*Sycophila foliatae*
28S		*Eurytoma* sp.	EU378340.1	98.151	
28S		*Sycophila* sp. 1	JN623675.1	98.231	
28S		*Sycophila* sp. 2	JN623675.1	98.257	
28S		*Sycophila* sp. 3	JN623675.1	98.192	
28S		*Sycophila* sp. 4	JN623675.1	99.022	*Sycophila texana*

Using the primer pair EurytomidF and HCO, we amplified a 501 base pair fragment of the “barcode” region of COI from between one and three specimens from each morpho‐group. These sequences all had high percentage matches to published sequences in GenBank (all ≥99%). Morpho‐group *Sycophila *sp. 1 had high percentage matches to sequences published from *Sycophila globuli* (99.01% and 100% similarity), morpho‐group *Sycophila* sp. 2 had high percentage matches to sequences published from *Sycophila flava *(all >99.6%), the specimen from morpho‐group *Sycophila *sp. 3 had a high percentage match to a sequence published from a specimen identified as *Sycophila *nr. *novascotiae *(100% similarity), and specimens from morpho‐group *Sycophila *sp. 4 had high percentage matches to sequences published from *Sycophila foliatae* (both ≥99.4%). Sequence matches for all amplified fragments of 28S and for the barcoding region of COI are presented in Table 1.

### 3.2 Population size and parasitism

Percent parasitism was significantly higher on Long Island (99.69% ± 0.014 *SE*) than on Cape Cod (55.14% ± 0.037 *SE*) (*F = *139.2; *df* = 160; *p* < 0.001; Table 2) in 2015. Parasitism remained steady between the years on Cape Cod, dropping only 1.3% in 2016 (*F* = 0.424, *df* = 163, *p* = 0.516). Long Island experienced a decline in parasitism (*F* = 25.07; *df* = 71; *p* < 0.001); however, this was due to the fact that the total number of individuals that emerged from branches collected on Long Island in 2016 was three individuals, one of which was a parasitoid in contrast to the 286 individuals collected in 2015 from the same trees (Table 2).

**Table 2:** Total percent parasitism of *Zapatella davisae* populations on Cape Cod, MA, and Long Island, NY, in 2015 and 2016

Region	Percent parasitism	Total No. of gall wasps	Total No. of parasitoids	Avg. No. of individuals^a^ per branch
2015				
Cape Cod	55.14	183	225	3.56 ± 0.36
Long Island	99.69	1	286	2.71 ± 0.37
2016				
Cape Cod	54.44	118	141	2.16 ± 0.31
Long Island	33.33	2	1	0.04 ± 0.02

a The term individuals includes the number of emerged gall wasps and parasitoids per branch.

The total number of parasitoids and gall wasps was used as an estimate of gall wasp establishment, because each parasitoid killed a previously developing gall wasp. Gall wasp establishment density (total number of parasitoids and gall wasps per branch) was significantly larger on Cape Cod than on Long Island for both 2015 and 2016 (z = 7.942; *df* = 1; *p* < 0.001) (Table 2). Population densities also declined overtime in both regions, causing a region × year interaction (*z* = −7.943; *df* = 1; *p* < 0.001; Table 2).

### 3.3 Relative contribution to total parasitism

There was a significant difference between the two regions in the proportion parasitized by each species in 2015 (χ^2^ = 92.782; *df* = 8; *p < *0.001; Table 3). Based on molecular data, all parasitoids present on Cape Cod were also present on Long Island, and Long Island contained an additional parasitoid, identified as a *Eurytoma* sp. (Hymenoptera: Eurytomidae). *Sycophila *nr. *novascotiae* contributed the most to parasitism on Long Island (in all cases of parasitism, 65% were due to *S. *nr. *novascotiae*) in 2015 (Table 3).

**Table 3:** Relative abundance of parasitoid species reared from *Zapatella davisae* on Cape Cod, MA, and Long Island, NY, in 2015 and 2016

Species	Morpho‐group	Species	2015		2016	
			Cape Cod (*n* = 225)	Long Island (*n* = 286)	Cape Cod (*n* = 141)	Long Island (*n* = 1)
1	Eurytoma sp.	*Eurytoma *sp.	0.0	1.7	–	–
2	Sycophila sp. 1	*Sycophila globuli*	2.9	3.0	–	–
3	Sycophila sp. 2	*Sycophila flava*	3.6	9.7	13.3	–
4	Sycophila sp. 3	*Sycophila nr*.* novascotiae*	34.1	65.0	40.0	1.0
5	Sycophila sp. 4	*Sycophila foliate*	57.3	19.8	42.6	–

## 4 DISCUSSION

For the oak gall wasps, a species‐rich group with a large community of parasitoids and inquilines (Stone et al., 2002), changes in geographical distribution and/or accidental introductions have been shown to result in outbreaks, sometimes as a function of enemy release (e.g. Cooper & Rieske, 2007; Eliason & Potter, 2000; Pujade‐Villar et al., 2014; Schönrogge et al., 1995). In the northeastern United States, *Z. davisae *outbreaks have caused extensive tree canopy decline and mortality (Buffington et al., 2016). Interestingly, tree damage and parasitism rates are regionally specific, with parasitism highest and defoliation lowest on Long Island. Parasitism rates were highly significant, yet in both locations, we found the parasitoid species emerging from *Z. davisae *galls to be the same, with the exception of one *Eurytoma* species.

Almost all of the recovered parasitoids are closely related members of the genus *Sycophila* (Hymenoptera: Eurytomidae), a genus of mostly endoparasitic koinobionts that attack gall wasp larvae or pupae (Gómeza, Nieves‐Aldrey, & Stone, 2013). All four *Sycophila* species are present in the United States, including S*ycophila globuli*,* S. flava*,* S. *nr. *novascotiae*, and *S. foliatae *(Peck, 1963). Most of these parasitoid species are naturally distributed throughout the eastern United States, with the exception of *S. globuli*, which has only been documented in the state of Illinois. As far as the other parasitoids are concerned, *Sycophila foliate* is commonly found in the southeastern United States, *S. nr. novascotiae *is present in the state of Virginia, and *S. flava* can be found throughout the United States, including the northeast (Peck, 1963). This parasitoid assemblage supports the theory that as a host species shifts its distribution, native predators will align themselves with the host species and build up densities over time (Didham et al., 2005; Sanders et al., 2003). It is likely that *Z. davisae* populations on Cape Cod experienced an outbreak due to a lag time with natural parasitoids in the region, whereas Long Island populations had already experienced this lag time in the 1990s, and as a result, the host population was controlled (Schönrogge et al., 1995).

Although the same Sycophila species were found in both Cape Cod and Long Island, their abundance was different in the two regions (Table 3). On Long Island, *S. *nr. *novascotiae* caused the highest level of parasitism. While this species was not as abundant on Cape Cod in 2015 as it was on Long Island, its abundance on Cape Cod increased slightly in 2016, suggesting that the population dynamics there may be converging with those on Long Island. It is very common for introduced insect parasitoid communities to converge with native communities with respect to species richness and abundance (Davis, 2009; Schönrogge et al., 1995). Since the *Z. davisae* parasitoid communities had similar richness in each region, only increases in abundance are needed for convergence.

In order to manage an insect pest, it is important to understand factors that influence its mortality and survival. It is unclear whether populations of *Z. davisae* will return to outbreaking densities in this region. Continuous monitoring of percent parasitism on Cape Cod should determine whether presently outbreaking populations will subside, as they did in our study site on Long Island in 2016 and more generally across Long Island in the 1990s (Pike et al., 2001). An understanding of how these infestations progress overtime can help predict the impact that *Z. davisae* will have on black oak populations in newly introduced areas in coastal New England and inform future integrated pest management efforts.

## AUTHORS’ CONTRIBUTIONS

MD, JCA, and JE conceived the ideas and designed methodology; MD collected the data; MD and JCA analyzed the data; All authors contributed critically to the drafts and gave final approval for publication.

## DATA ACCESSIBILITY

The *Sycophila* sequences were recently added to the manuscript and will be submitted to GenBank as soon as possible. They will be added to the MS immediately after they are posted to GenBank. Previous sequences of *Z. davisae* can be found under GenBank Accession Number: KU567186.1.
